# Supplementary material for: Time Spent on Daily Activities and Its Association with Life Satisfaction among Czech Adolescents from 1992 to 2019
Source: Int J Environ Res Public Health. 2022 Aug 1;19(15):9422. doi: 10.3390/ijerph19159422 (PMC9367733; doi:10.3390/ijerph19159422)
Supplement: Supplementary file 1 [file ijerph-19-09422-s001.zip › ijerph-1754432-supplementary.pdf]

Table S1

Linear regression with splines predicting life satisfaction based on time spent sleeping (controlling for cohort, gender, age, and parents' earnings)

Predictors	*B*	SE_B	95% CI_B		*p*	*B*	SE_B	95% CI_B		*p*
Intercept	3.02	0.02	2.97	3.06	<.001	2.68	0.08	2.52	2.83	<.001
Cohort: 2011	−0.07	0.03	−0.12	−0.02	.006	0.06	0.04	−0.01	0.14	.092
Cohort: 2001	−0.02	0.03	−0.08	0.03	.425	0.02	0.04	−0.05	0.10	.538
Cohort: 1992	−0.08	0.03	−0.14	−0.02	.008	0.10	0.03	0.04	0.16	<.001
Gender: Female	−0.08	0.02	−0.11	−0.04	<.001	−0.07	0.02	−0.11	−0.04	<.001
Age: ≥ 16 years	0.02	0.02	−0.01	0.06	.213	0.04	0.02	0.00	0.08	.060
Earn: less	−0.18	0.03	−0.23	−0.13	<.001	−0.17	0.03	−0.22	−0.12	<.001
Earn: more	0.09	0.02	0.04	0.14	<.001	0.10	0.02	0.05	0.14	<.001
Sleep 0–7 h						0.03	0.01	0.01	0.05	.012
Sleep 7–8 h						0.09	0.03	0.04	0.15	<.001
Sleep 8–9 h						−0.02	0.03	−0.09	0.04	.468
Sleep 9–12.5 h						−0.04	0.03	−0.09	0.02	.238
R^2			.043					.056		
Adjusted R^2			.041					.052		
ΔR^2			–					.013		
$F(df)$ for R^2			16.88 (7, 2606), $p < .001$					13.97 (11, 2597), $p < .001$		
$F(df)$ for ΔR^2			–					9.08 (4, 2597), $p < .001$		

Note. The reference category for cohort is the last (2019) cohort and the reference category for parents' earnings is "My parents earn about the same as other parents". The coefficients for Sleep are partial regression slopes for different amounts of time spent sleeping (in hours).

Table S2

Linear regression with splines predicting life satisfaction based on time spent commuting to school (controlling for cohort, gender, age, and parents' earnings)

Predictors	B	SE_B	95% CI_B		p	B	SE_B	95% CI_B		p
Intercept	3.02	0.02	2.97	3.06	<.001	2.92	0.06	2.81	3.04	<.001
Cohort: 2011	–0.07	0.03	–0.12	–0.02	.006	0.06	0.04	–0.01	0.14	.111
Cohort: 2001	–0.02	0.03	–0.08	0.03	.425	0.02	0.04	–0.05	0.09	.613
Cohort: 1992	–0.08	0.03	–0.14	–0.02	.008	0.08	0.03	0.02	0.14	.010
Gender: Female	–0.08	0.02	–0.11	–0.04	<.001	–0.08	0.02	–0.12	–0.04	<.001
Age: ≥ 16 years	0.02	0.02	–0.01	0.06	.213	0.02	0.02	–0.02	0.06	.338
Earn: less	–0.18	0.03	–0.23	–0.13	<.001	–0.18	0.03	–0.23	–0.13	<.001
Earn: more	0.09	0.02	0.04	0.14	<.001	0.08	0.02	0.03	0.13	<.001
Commute: 0–0.5 h						0.05	0.12	–0.18	0.28	.680
Commute: 0.5–1 h						–0.01	0.05	–0.11	0.09	.830
Commute: 1–5 h						–0.01	0.02	–0.04	0.03	.787
R^2			.043					.043		
Adjusted R^2			.041					.039		
ΔR^2			–					<.001		
$F(df)$ for R^2			16.88 (7, 2606), p < .001					10.29 (10, 2302), p < .001		
$F(df)$ for ΔR^2			–					0.10 (3, 2302), p = .960		

Note. The reference category for Cohort is the last (2019) cohort and the reference category for parents' earnings (Earn) is "My parents earn about the same as other parents". The coefficients for Commute are partial regression slopes for different amounts of time spend commuting to/from school (in hours).

Table S3

Linear regression with splines predicting life satisfaction based on time spent at school (controlling for cohort, gender, age, and parents' earnings)

Predictors	*B*	SE_B	95% CI_B		*p*	*B*	SE_B	95% CI_B		*p*
Intercept	3.02	0.02	2.97	3.06	<.001	2.85	0.10	2.66	3.04	<.001
Cohort: 2011	–0.07	0.03	–0.12	–0.02	.006	0.06	0.04	–0.01	0.14	.095
Cohort: 2001	–0.02	0.03	–0.08	0.03	.425	0.01	0.04	–0.07	0.08	.836
Cohort: 1992	–0.08	0.03	–0.14	–0.02	.008	0.08	0.03	0.02	0.14	.011
Gender: Female	–0.08	0.02	–0.11	–0.04	<.001	–0.08	0.02	–0.11	–0.04	<.001
Age: ≥ 16 years	0.02	0.02	–0.01	0.06	.213	0.03	0.02	–0.01	0.06	.169
Earn: less	–0.18	0.03	–0.23	–0.13	<.001	–0.18	0.03	–0.23	–0.13	<.001
Earn: more	0.09	0.02	0.04	0.14	<.001	0.09	0.02	0.05	0.14	<.001
School: 0–5 h						0.02	0.02	–0.02	0.06	.382
School: 5–6 h						0.01	0.03	–0.06	0.07	.813
School: 6–7 h						–0.02	0.03	–0.08	0.04	.550
School: 7–11.5 h						0.01	0.03	–0.05	0.06	.852
R^2	.043					.043				
Adjusted R^2	.041					.039				
ΔR^2	–					<.001				
$F(df)$ for R^2	16.88 (7, 2606), $p < .001$					10.59 (11, 2571), $p < .001$				
$F(df)$ for ΔR^2	–					10.59 (4, 2571), $p = .842$				

Note. The reference category for Cohort is the last (2019) cohort and the reference category for parents' earnings (Earn) is "My parents earn about the same as other parents". The coefficients for School are partial regression slopes for different amounts of time spent at school (in hours).

Table S4

Linear regression with splines predicting life satisfaction based on time spent playing sports or exercising (controlling for cohort, gender, age, and parents' earnings)

Predictors	*B*	SE_B	95% CI_B		*p*	*B*	SE_B	95% CI_B		*p*
Intercept	3.02	0.02	2.97	3.06	<.001	2.90	0.03	2.83	2.96	<.001
Cohort: 2011	–0.07	0.03	–0.12	–0.02	.006	0.07	0.04	–0.01	0.14	.075
Cohort: 2001	–0.02	0.03	–0.08	0.03	.425	0.01	0.04	–0.06	0.08	.768
Cohort: 1992	–0.08	0.03	–0.14	–0.02	.008	0.08	0.03	0.02	0.14	.009
Gender: Female	–0.08	0.02	–0.11	–0.04	<.001	–0.07	0.02	–0.10	–0.03	<.001
Age: ≥ 16 years	0.02	0.02	–0.01	0.06	.213	0.03	0.02	0.00	0.07	.084
Earn: less	–0.18	0.03	–0.23	–0.13	<.001	–0.18	0.03	–0.23	–0.13	<.001
Earn: more	0.09	0.02	0.04	0.14	<.001	0.09	0.02	0.04	0.13	<.001
Sport: 0–1 h						0.06	0.03	0.01	0.12	.014
Sport: 1–2 h						0.01	0.04	–0.06	0.08	.750
Sport: 2–5.8 h						0.01	0.03	–0.04	0.06	.767
R^2	.043					.049				
Adjusted R^2	.041					.046				
ΔR^2	–					.006				
$F(df)$ for R^2	16.88 (7, 2606), $p < .001$					13.48 (10, 2591), $p < .001$				
$F(df)$ for ΔR^2	–					5.35 (3, 2591), $p = .001$				

Note. The reference category for Cohort is the last (2019) cohort and the reference category for parents' earnings (Earn) is "My parents earn about the same as other parents". The coefficients for Sport are partial regression slopes for different amounts of time spend playing sports or exercising (in hours).

Table S5

Linear regression with splines predicting life satisfaction based on time spent playing online games (controlling for gender, age, and parents' earnings)

Predictors	*B*	SE_B	95% CI_B		*p*	*B*	SE_B	95% CI_B		*p*
Intercept	2.99	0.02	2.95	3.03	<.001	3.03	0.03	2.97	3.09	<.001
Gender: Female	–0.07	0.02	–0.10	–0.03	<.001	–0.07	0.02	–0.12	–0.03	.002
Age: ≥ 16 years	0.03	0.02	–0.01	0.06	.147	0.02	0.02	–0.03	0.07	.476
Earn: less	–0.18	0.03	–0.23	–0.14	<.001	–0.18	0.03	–0.24	–0.11	<.001
Earn: more	0.09	0.02	0.05	0.14	<.001	0.11	0.03	0.05	0.16	<.001
Gaming: 0–1 h						0.02	0.03	–0.04	0.08	.588
Gaming: 1–11 h						–0.03	0.01	–0.05	–0.01	<.001
R^2	.037					.043				
Adjusted R^2	.035					.040				
ΔR^2	–					.008				
$F(df)$ for R^2	16.64 (4, 1728), $p < .001$					12.99 (6, 1718), $p < .001$				
$F(df)$ for ΔR^2	–					6.77 (2, 1718), $p = .001$				

Note. The reference category for parents' earnings (Earn) is "My parents earn about the same as other parents". The coefficients for online gaming are partial regression slopes for different amounts of time spend playing online games (in hours).
